# Emerging Themes of Global Dust Research: Linking Physical Processes to Human Health, Safety, and Welfare

**DOI:** 10.1029/2025GH001739

**Published:** 2026-03-16

**Authors:** Karin Ardon‐Dryer, Daniel Tong, Junran Li

**Affiliations:** ^1^ Department of Geosciences Atmospheric Science Group Texas Tech University Lubbock TX USA; ^2^ Center for Satellite and Earth Science Research and Department of Atmospheric Oceanic and Earth Sciences George Mason University Fairfax VA USA; ^3^ Department of Geography The University of Hong Kong Hong Kong SAR China

**Keywords:** dust research, DANA

## Abstract

Dust and dust storms are known to negatively affect human health, safety, and welfare. Most dust‐related studies have previously focused on the physical processes of dust initiation, transport, and deposition at various temporal and spatial scales. In recent years, more scholars have attempted to link the physical processes of dust to human health, safety, and welfare. The idea for this special collection, named “Dust and dust storms, from physical processes to human health, safety, and welfare” was proposed by members of the Dust Alliance for North America (DANA), a partnership of scientists and practitioners with the purpose of accelerating the transition of dust‐related research into service. Papers in this special issue covered a wide range of topics, including the physical processes of dust particles and dust events, and their subsequent impacts on human health, transportation safety, and welfare, with various temporal and spatial scales. These papers were jointly published in three American Geophysical Union (AGU) journals, namely, GeoHealth, Earth's Future, and Journal of Geophysical Research (JGR)‐Atmospheres. The special collection opened in May 2023 and closed in June 2025. Eventually, a total of 24 papers were published as part of this collection, including five papers in GeoHealth, two in Earth's Future, and 17 in the JGR‐Atmospheres. Collectively, these papers report the latest interdisciplinary efforts by the research community to understand the physical processes of dust and its societal effects.

## Plain Language Summary

Dust in the air can harm people's health, safety, and daily lives. In the past, most dust research focused on the physical processes: how and where it starts, travels, and settles. More recently, scientists have worked to connect these physical processes to societal impacts, such as health problems and traffic hazards. To support this effort, the Dust Alliance for North America (DANA) organized this special collection to explore the science of dust and how it affects humans and the environment. This collection, published between May 2023 and June 2025, brought together 24 studies on these topics across three journals (GeoHealth, Earth's Future, and the Journal of Geophysical Research–Atmospheres).

## Key Points

- Dust and dust storms are known to negatively affect human health, safety, and welfare
- The Dust Alliance for North America organized this collection to explore science of dust and how it affects humans and the environment
- 24 studies published in this special collection across three AGU journals (GeoHealth, Earth's Future, and the JGR‐Atmospheres)

## 1 Introduction

Human exposure to dust particles has been associated with various adverse health effects (Lwin et al., 2023; Tong, Gill, et al., 2023). Besides human health, dust also affects environmental health by providing nutrients that increase phytoplankton biomass, contaminating water supply and food, spreading pathogens and marine organisms, causing Valley Fever among domestic and wild animals, transporting heavy metals and radionuclides, and reducing solar power generation (Tong, Gill, et al., 2023). Dust storms are also well documented as a safety hazard to road transportation, aviation, and marine navigation (Li et al., 2018; Nickovic et al., 2021; Tong, Feng, et al., 2023). This paper summarizes and highlights the main findings of papers published as part of a special dust focus collection named “Dust and dust storms, from physical processes to human health, safety, and welfare.” This collection invited submissions that focus on both natural and man‐made dust events, that examine blowing dust events of different scales, from dust devils to dust storms from a multidisciplinary perspective, including but not limited to remote sensing, particle toxicology, geochemistry, social sciences using clinical medicine, epidemiological, in vivo and in vitro studies, that enhance the knowledge on dust and their health implications.

The idea for this special collection was proposed by members of the Executive Committee of the Dust Alliance for North America (DANA; https://dustalliance.org). The DANA is a partnership of scientists and practitioners with the purpose of accelerating the transition of dust‐related research into service (Sprigg et al., 2022).​ Although the focus is on North America, DANA's mission is to inspire, support, and promote global science and service collaboration to gain and adapt knowledge of airborne dust particulates to mitigate health, safety, and quality of life risks. Papers in this special collection represent a joint American Geophysical Union (AGU) collection with three journals, JGR‐Atmospheres, GeoHealth, and Earth's Future. The special collection ran for two years from May 2023 to June 2025. Eventually, of the collection totaled 24 papers. Five papers were published in the journal GeoHealth, two in the journal Earth's Future, and 17 in the JGR‐Atmospheres.

Studies in this collection investigated dust from across the world, including Asia (Chandel et al., 2025; Pi et al., 2025; Xi, 2023; Zhang et al., 2025), the Middle East (Karumuri et al., 2024; Mostamandi et al., 2023), Africa (Pons et al., 2025), the North America (Miller & Ramseyer, 2024), dust that travels from Africa to north America (Kahn & Limbacher, 2025; Miller & Ramseyer, 2024), and more specifically dust from the United States (Ardon‐Dryer et al., 2023; Chappell et al., 2023; Hohsfield et al., 2023; Khatei et al., 2024; Dhital et al., 2024; McKenna Neuman et al., 2024; Reynolds et al., 2024; Reynolds et al., 2025; Ardon‐Dryer and Aziz, 2025; Norris et al., 2025).

Several studies analyzed specific dust events (e.g., Dhital et al., 2024; Xi, 2023), while others examined longer periods with multiple dust events (Ardon‐Dryer and Aziz, 2025; Karumuri et al., 2024; Pi et al., 2025). Several of the papers investigated the meteorological conditions that initiate dust events (Dhital et al., 2024; Pons et al., 2025; Xi, 2023) or the chemical properties of the dust particles (Putman et al., 2025; Reynolds et al., 2025; Zhang et al., 2025), or the impact of dust events on human health (Hohsfield et al., 2023; Ramirez‐Diaz et al., 2025), and air quality (Ardon‐Dryer and Aziz, 2025; Ardon‐Dryer et al., 2023; Putman et al., 2025). Also, a variety of tools were used to examine the dust events. Some examined dust in the laboratory settings (Putman et al., 2025; Ramirez‐Diaz et al., 2025; Reynolds et al., 2024), whereas others used experimental tools like wind tunnels (Khatei et al., 2024; McKenna Neuman et al., 2024). Several papers used models to study the dust (e.g., Reid et al., 2025), such as the WRF‐Chem (Dhital et al., 2024; Karumuri et al., 2024; Mostamandi et al., 2023), others examined atmospheric measurements that were measured during the dust events (Ardon‐Dryer and Aziz, 2025; Putman et al., 2025).

Two papers (Cui et al., 2025; Zhang et al., 2024) were published in Earth's Future. Cui et al. (2025) explored how changes in particle size affect the optical properties and local aerosol‐radiation interactions, which were previously overlooked amidst significant shifts in emission behaviors. They found that increased coarse particles enhance fine particle coagulation, contributing to higher coarse particle levels and a reduction in coarse particle peak size. This change helped scatter more sunlight, partially offsetting the warming effect. The paper by Zhang et al. (2024) analyzed wind erosion change in Africa during 2001–2020 using the Revised Wind Erosion Equation (RWEQ). The authors found that the magnitude of wind erosion fluctuated with an overall decreasing trend of wind erosion, but this trend was not uniform among regions with different aridity levels. Both wind speed and vegetation coverage were found to be critical to the fluctuation of wind erosion in Africa.

A total of five papers were published in GeoHealth. Two of these papers focused on the air quality aspect of dust. In Ardon‐Dryer et al. (2023), the authors found that dust events in the United States were not often prioritized in air‐quality‐mitigation‐strategies. They argued that several factors (provided in the paper) contribute to the underestimation of the social and environmental impacts of dust events. The study by Ardon‐Dryer and Aziz (2025) provided insight into the problem raised by Ardon‐Dryer et al. (2023) with specific examples of short‐duration convective dust events from the greater Phoenix area in Arizona, United States. They stated that these short‐duration dust events can degrade the air quality and pose a potential health threat to millions of people. The authors argued that there is a need to consider shorter time intervals (<10 min) to capture the PM concentrations accurately and highlight the importance of real‐time monitoring and accurate characterization of short‐duration events to assess their impacts on air quality and human health.

Hohsfield et al. (2023) and Ramirez‐Diaz et al. (2025) papers in GeoHealth focused on the health impact of dust. Hohsfield et al. (2023) attempted to identify appropriate dust data for large‐scale population epidemiological studies. They compared the accuracy of four publicly available data products to determine the most suitable one, which could examine the health impact of dust storms in the southwestern United States. Ramirez‐Diaz et al. (2025) examined the impact of five clay minerals on human epithelial alveolar cells utilizing the Single‐Cell Analysis (in vitro). The authors reported that Ca‐rich montmorillonite caused a significant increase in cell death and a decrease in cell proliferation compared to kaolinite, but both clays had a similar impact on the type of death (necrosis replaced apoptosis as the primary mechanism for cell death).

In another study published in GeoHealth, Putman et al. (2025) analyzed physical and geochemical properties of dust samples collected at 17 sites during fall 2022 across northern Utah (United States). The physical and geochemical properties of the samples were used to quantify the presence of metals and their health impact. Among the 11 priority pollutant metals examined, the study found that Tl, As, Pb, Co, and Cr contributed most to the estimated health hazard, where As and Pb were likely derived from emissions from the Salt Lake playa.

The 17 papers that were published in the JGR‐Atmospheres cover a wide scope of dust‐related topics. For instance, Zhang et al. (2025) provided a comprehensive analysis of the morphology and mineral composition of dust particles generated by sandblasting from Gobi soils and sand dunes of the Tengger and the Ulan Buh Deserts in northern China. Differences in mineral compositions and particle sizes of each of these sources enabled the assessment of their effects on atmospheric chemistry and solar radiation transfer. Reynolds et al. (2025) identified light‐absorbing particles in 38 snow samples (2013–2016) from the mountainous Upper Colorado River basin by performing laboratory analysis that measured spectral reflectance, chemical, physical, and magnetic properties. Different types of particles were found, including carbonaceous matter, black road tire wear particles, as well as dark rock and mineral particles. Norris et al. (2025) also collected dust deposition samples (from 2019 to 2022) at six sites on San Jacinto Peak in Southern California. Dust chemical compositions vary among sites and seasons, indicating that these sites receive dust from multiple sources throughout the year. The authors argued that the chemical variation in the dust was caused by inputs from regional dust source areas such as the Mojave Desert and urban or industrial particulate matter. The radiative effects of atmospherically deposited microplastics from road‐tire wear were elucidated by Reynolds et al. (2024). Black tire particles were identified in Colorado Rocky Mountain snow cover using techniques of two‐dimensional gas chromatography, energy‐dispersive spectroscopy under a scanning electron microscope, and stereomicroscopy. This paper also estimated the mass of tire‐wear particles produced at the tire‐road interface during 2023 at 6.55 M tonnes, a proportion of which (ultimately as much as 10%–30%) became airborne.

Both Khatei et al. (2024) and McKenna Neuman et al. (2024) examined the generation of dust particles using a wind tunnel. Khatei et al. (2024) quantified the effects of soil salinity on the threshold velocity for wind erosion and dust production in dry soils with different textures treated with salt‐enriched water at different concentrations. They found that even though saline soil showed resistance to wind erosion in the absence of abraders, salt crusts were readily ruptured by saltating sand grains, resulting in comparable or even higher particulate matter emissions compared to non‐saline soils. McKenna Neuman et al. (2024) used playa sediment from Owens Lake in California, USA, in wind tunnel experiments to examine the rate of comminution during transport, the role of bed roughness, humidity, system dynamics, and the proportionate amount of dust production. They found that particle aggregates formed from the playa sediment were rapidly fractured during the process of transport, and fractured particle aggregates produced less than 4% of the suspended PM_10_. Dust suspension was highly sensitive to secondary flow structures within the turbulent wind field. Reid et al. (2025) used a computer simulation to model the electric and magnetic fields that form inside a dust devil. Their finding offered a useful framework for dust devils, particularly for estimating field strengths based on observed signatures and geometry. An approach that will contribute to a broader understanding of dust‐related electromagnetic effects, especially in environments where they pose risks to instrumentation and operations.

Several papers, for example, Chappell et al. (2023), Mostamandi et al. (2023), and Karumuri et al. (2024), focused on the optical and radiative impact of dust particles and dust events. Chappell et al. (2023) compared measurements of dust optical depth frequency of occurrence (probability), and satellite‐observed, dust‐emission‐frequency from point sources across North America. They found up to a two orders of magnitude difference between the probability of dust optical depth and point sources. They argued that models overestimated the dust‐emission probability but showed strong spatial relations to dust‐point sources. They also pointed out that the traditional dust emission model overestimated large dust emissions over vast vegetated areas and produced considerable inaccurate changes in dust emissions. Mostamandi et al. (2023) evaluated the radiative dust effect and deposition rates in the Middle East using the free‐running WRF‐Chem model. They found that incorporating coarse dust in model simulations and data assimilation improved the overall accuracy of the dust mass balance and its impact on environmental systems and solar devices. Karumuri et al. (2024) studied the impact of dust on radiation over the Arabian Peninsula during the reported high, low, and normal dust seasons (March–August) of 2012, 2014, and 2015 (respectively) using the WRF‐Chem model. The study found that the maximum dust concentrations over the dust‐source regions reach vertically up to 700 hPa (∼3,000 m) during the high dust season, but only up to 900–950 hPa (approximately 500–100 m) during the low/normal dust seasons.

Several additional papers, that is, Xi (2023), Dhital et al. (2024), Chandel et al. (2025), and Pi et al. (2025), examined specific dust events or periods with multiple dust events. Xi (2023) explored the geomorphic, meteorological, and hydroclimatic drivers of unusual dust activity with an extreme salt storm from the Aralkum desert that impacted large parts of central Asia during early summer 2018. The study found that the dust outbreaks were triggered by recurrent cold air invasion from high latitudes in association with a strong high‐pressure system over western Russia and slow‐moving planetary waves in the upper troposphere. Dhital et al. (2024) investigated the large‐scale meteorological conditions that led to two dust events on the Colorado Plateau and in the northern Chihuahuan Desert in the United States. The authors found that polar and subtropical jet stream interaction was a common upper‐level meteorological feature before these dust events. Chandel et al. (2025) used satellite observations to identify conditions that lead to dust‐loading events over the Indian Himalayas. The study identified different transport trajectories from different source regions: (a) A long‐range transport, in which mineral dust particles from western Pakistan and the Middle East were dominant, and (b) a short‐range transport, in which the dust originating from the arid western regions of the Indian subcontinent carried pollutants, having traveled across the heavily polluted areas of North India before reaching the Himalayas. In addition, Pi et al. (2025) analyzed the spatiotemporal characteristics of dust events recorded in 1941 meteorological stations in China, from 1960 to 2020. They found that dust events decreased significantly from 1960 to 2020. The study showed that the frequency of dust events was two orders of magnitude greater in the cold desert climate compared with the other climate zones, even though the annual wind speed was not always greater. Dust activities in China were associated with meteorological parameters and the impact of human activities (e.g., overcultivation), which seems to be more significant in non‐dry climate zones.

Finally, Pons et al. (2025), Miller and Ramseyer (2024), and Kahn and Limbacher (2025) examined the influence of meteorological conditions in the Sahara area on the generation of dust events and the transport of dust over Europe and across the Atlantic toward the Caribbean region. Pons et al. (2025) found that a Saharan dust outbreak (March 2014) coincided with intense precipitation in western Iberia. From this observation, they surmised that, over the past four decades, changes in weather patterns caused by changes in climate have increased dust loading in the central‐eastern Mediterranean. Miller and Ramseyer (2024) employ a self‐organizing map classification to identify the common trans‐Atlantic dust transport typologies associated with the Saharan Air Layer during June and July 1981–2020. They found that days with integrated dust transport patterns demonstrate island‐wide negative precipitation anomalies in Puerto Rico. Kahn and Limbacher (2025) examined the June 2020 trans‐Atlantic dust transport using the NASA Earth Observing System's Multi‐angle Imaging Spectro Radiometer (MISR) instrument. The study showed that MISR is able to monitor changes in particle properties, opening the possibility of using satellite‐derived dust‐plume evolution to evaluate long‐range transport of dust.

In summary, this special collection of 24 papers, jointly published by the journals of GeoHealth, Earth's Future, and Journal of Geophysical Research‐Atmospheres, represents a wide range of authorship and study areas that include Asia, Africa, Europe, and North America. Although these studies used a myriad of methodologies and data sets, they have shared concerns on linking the physical processes of dust and dust events to human health, safety, and overall welfare, an emerging field of dust research in the coming decades.

## Conflict of Interest

The authors declare no conflicts of interest relevant to this study.

## Data Availability

All the information presented can be found in the collection “Dust and dust storms, from physical processes to human health, safety, and welfare.” No data was used or generated as part of this paper.
